# HLA Class III: A susceptibility region to systemic lupus erythematosus in Tunisian population

**DOI:** 10.1371/journal.pone.0198549

**Published:** 2018-06-18

**Authors:** Hend Hachicha, Nadia Mahfoudh, Hajer Fourati, Nesrine Elloumi, Sameh Marzouk, Sawsan Feki, Raouia Fakhfakh, Faten Frikha, Abir Ayadi, Amira Maatoug, Lilia Gaddour, Feiza Hakim, Zouheir Bahloul, Hafedh Makni, Hatem Masmoudi, Arwa Kammoun

**Affiliations:** 1 Immunology Department, Habib Bourguiba University Hospital, Sfax, Tunisia; 2 UR12SP14, Habib Bourguiba University Hospital, Sfax, Tunisia; 3 Faculty of Medicine of Sfax, University, Sfax, Tunisia; 4 Histocompatibility Department, Hedi Chaker University Hospital, Sfax, Tunisia; 5 Internal Medicine Department, Hedi Chaker University Hospital of Sfax, Sfax, Tunisia; Centro Cardiologico Monzino, ITALY

## Abstract

**Background and objectives:**

Short tandem repeats (STR) are usually used as informative polymorphic markers for genetic mapping and for disease susceptibility analysis. The involvement of these microsatellite markers localized in the MHC region was reported in many auto-immune diseases.

In this study we analyzed for the first time eight polymorphisms of microsatellite loci at the HLA region: D6S291, D6S273, TNFa, b and c, MICA, D6S265 and D6S276, in Tunisian systemic lupus erythematosus (SLE) patients.

**Materials and methods:**

We performed a case control study in which the microsatellite loci were amplified using specific primers labeled with NED, VIC, PET or 6-FAM and analyzed using GeneScan software 3.7. For the statistical analysis, we used SPSS software and we performed a sub-haplotype scoring test using the haplo.stats software developed in the R language.

**Results:**

We found that two mean associated regions existed; the most statistically significant encompassed the 3 TNF markers (p = 0.0003, OR = 19.34); the latter covered the DR region. In fact, when scoring haplotypes in 3 marker- sliding windows, the p value increased as we moved away from the TNF region and decreased again when we approached the DRB1 locus. We also established for the first time the negative association between alleles of D6S291 and SLE. The majority of clinical and serological correlations were noted with TNF alleles.

**Conclusion:**

Our results confirm the association between TNF and DRB1 polymorphisms and SLE. The association between alleles of D6S291 and SLE needs however to be verified by the analysis of other markers beyond this region.

## 1. Introduction

The chromosomal region including the human leukocyte antigen (HLA) Class II to Class I genes has been implicated in susceptibility to systemic lupus erythematosus (SLE) and other autoimmune diseases such as rheumatoid arthritis, insulin-dependent diabetes mellitus, pemphigus foliaceus, and Gougerot Sjogren’s syndrome [1–2–3]. This region situated on the short arm of chromosome six encodes several molecules: HLA Class I and Class II implicated in the presentation of peptides to the immune cells which is an essential phenomenon for the production of auto-antibodies characterizing auto-immune diseases; it also encodes other proteins suggested to be implicated in the physiopathology of this kind of diseases for instance complement fractions and TNF[4].

In SLE, the strongest genetic association described so far, has been with the HLA class II alleles. Thus, susceptibility to SLE has been correlated specially with HLA-DRB1 and HLA-DQB1 alleles in different ethnic groups [5–6]. In our population, we demonstrated a predisposition factor effect of HLA DRB1*03 and HLA DRB1*15 for SLE[7]; this was in accordance with the majority of genetic studies.

Short tandem repeats (STR)are usually used as informative polymorphic markers for genetic mapping and for disease susceptibility analysis. The involvement of these microsatellite markers localized in the MHC region was reported in many auto-immune diseases[2, 8,9].Several data have reported the association of HLA microsatellite with SLE[10].

In this study we analyzed eight polymorphisms of microsatellite loci at 6p21.3–21.4 spanning HLA region: D6S291, D6S273, TNFa, b and c, major histocompatibility complex class I chain-related gene A (MICA), D6S265 and D6S276, in 87 SLE patients compared to 123 healthy individuals recruited from the south of Tunisia and this in order to investigate any eventual new susceptibility or prognostic markers of SLE.

## 2. Materials and methods

### 2.1. Patients and controls

We performed a case /control study during five years (January 2012- January2017). This case-control study was approved by the Ethics committee of the university hospitals of Sfax, Tunisia. We recruited 210 individuals (87 patients with SLE and 123 healthy controls) originating from the South of Tunisia. Patients and controls gave their written informed consent.

Patients included in this study fulfilled the American College of Rheumatology (ACR) criteria for the diagnosis of SLE. An exhaustive information sheet containing clinical and serological features was filled for each patient.

### 2.2. Genotyping methods

We extracted genomic DNA from ethylene diamine tetra-acetic acid (EDTA) peripheral blood using a phenol/chloroform technique. We conducted an association study using 8 microsatellite polymorphic markers: D6S291, D6S273, TNFa, b and c, MICA, D6S265 and D6S276 covering the HLA class I, III and II regions (S1 Fig).

The microsatellite loci were amplified using specific primers (Table 1) determined from an NCBI db MHC database (http://www.ncbi.nlm.nih.gov/gv/mhc/xslcgi.cgi?cmd=mssearch) and provided by Perkin Elmer (Applied Biosystems^®^, CA,USA).Forward primers were labeled with NED, VIC, PET or 6-FAM fluorescent labels. Amplified products were run on ABI prism 310 DNA sequencer (Perkin–Elmer^®^, CT, USA). The output file was analyzed using GeneScan software 3.7.

**Table 1:** Characteristics of studied STR.

*STR*	*Localization*	*Repeats*	*Primers*	*Labeling*
***D6S291***	6p21.2 ; 3,6-4cM cent DPB1	CA	F:5’-CTCAGAGGATGCCATGTCTAA-3’ R:5’-GGGGATGACGAATTATTCACTAACT-3’	6-FAM-
***D6S273***	Hsp70-Bat2 (96 kb) Tel Hsp70	CA	F:5’-GCAACTTTTCTGTCAATCCA-3’ R:5’-ACCAAACTTCAAATTTTCGG-3’	6-FAM-
***TNFc***	Intron of TNFB	TC	F:5’-GGTTTCTCTGACTGCATCTTGTCC-3’ R:5’-TCATGGGGAGAACCTGCAGAGAA-3’	PET
***TNFa***	6p21.3 Tel (3,5 kb)/TNF B	AC	F:5’-GCCTCTAGATTTCATCCAGCCACA-3’ R:5’-CCTCTCCCCCTGCAACACACA-3’	VIC
***TNFb***	6p21.3 Tel. (3,5 kb)/TNF B	TC	F:5’-GCACTCCAGCCTAGGCCACAGA-3’ R:5’-GTGTGTGTTGCAGGGGAGAGAG-3’	VIC
***MICA***	40 Kb cent to HLA-B	GCT	F:5’-CCTTTTTTTCAGGGAAAGTGC-3’ R:5’-CCTTACCATCTCCAGAAACTGC-3’	NED
***D6S265***	HLA-E/HLA-A	CA	F:5’-ACGTTCGTACCCATTAACCT-3’ R:5’-ATCGAGGTAAACAGCAGAAA-3’	NED
***D6S276***	Tel (6500 kb) HLA-A	CA	F:5’-TCAATCAAATCATCCCCAGAAG-3’ R:5’-GGGTGCAACTTGTTCCTCCT-3’	VIC

### 2.3. Statistical study

We first compared for each marker the global alleles distribution between patients and healthy controls, using the BIGDAWG package implemented in R (https://cran.r-project.org/web/packages/BIGDAWG).

For detailed analysis, we used the Statistical Software for The Social Sciences(SPSS version 20.0, USA). We first compared STR frequencies in SLE patients and healthy controls; then we tested the impact of each allele on the different clinical and serological disease features. We searched for significant correlations using the χ2 test. P values were considered as statistically significant if < 0.05. Significant p-values were corrected(pc) by the number of alleles tested for each marker (Bonferroni’s corrections) and pc <0.05 was considered statistically significant. The odds ratio (OR) and its 95% confidence interval (CI) were calculated for each allele to estimate the magnitude of the association.

We also used binary logistic regression to assess the real contribution of STR polymorphism at the occurrence of SLE, and to see if the associations found are due to a linkage disequilibrium (LD) with HLA class II alleles already demonstrated to be associated with SLE in our population [7].

To assess the association between haplotypes and SLE, analytical approaches implemented in the software package Haplo.stats (http://cran.r-project.org/web/packages/haplo.stats) were used. The software uses an expectation-maximization algorithm to infer haplotypes from the observed genotypes with an unknown linkage phase.

In this analysis, we evaluated the association of sub-haplotypes with SLE, in a sliding window of 3 loci. Given an ordered set of markers from 1 to 8, sliding windows of overlapping haplotypes are tested in sequence, thereby markers 1–2–3 are treated as a single haplotype, then markers 2–3–4 are treated as a single haplotype, then markers 3–4–5, etc.

Sub-haplotype analysis included DRB1* and DQB1* loci reported in our previous study [7].

## 3. Results

### 3.1. Demographic, clinical and serological characteristics

The age in our SLE patients ranged from 14 to 90 years (mean age = 32.11± 13.57). The sex ratio female patients to male patients (F/M) was 6.9 (Table 2). The control group was formed by 72 women and 51 men. Their average age was 32 years ± 9.28.

**Table 2:** Clinical and immunological manifestations in our patients.

	Number	Frequency%	Availability
**Sex F/M**	76/11		
**Clinical manifestations**			
Malar rush	41	51.9	90.8
Photosensitivity	36	45.6	90.8
Buccal ulceration	11	13.9	90.8
Anemia	66	48.6	89.7
Arthritis	18	22.8	90.8
Polyathralgia	51	64.6	90.8
Lupus nephritis	38	48.1	90.8
Pericarditis	19	24.1	90.8
Pleurisy	12	15.2	90.8
Raynaud's syndrome	6	7.6	90.8
Thrombosis	13	17.1	87.4
Neurologic disorders	12	16.2	85.1
**Serology**			
Anti-dsDNA	60	70.6	97.7
Anti-nucleosome	51	60.7	96.6
Anti-Sm	25	29.8	96.6
Anti-RNP	25	29.8	96.6
Anti-SSA	48	57.1	96.6
Anti-SSB	20	23.8	96.6
Anti-Ribosome	16	19	96.6
Anti-Histone	27	32.1	96.6
Anti RO52	31	36.9	96.6
Low CH50	36	60	69
Low C3	31	46.3	77
Low C4	34	50.7	77
Anti-cardiolipin	42	60	80.5
Anti-β2gpI	23	35.9	73.6
Rheumatoid factors	14	24.6	65.5

F: female, M: male.

### 3.2. HLA microsatellites alleles

Microsatellite allele distributions were different in patients and controls. The difference was significant for 2 loci: TNFc (p = 10^−6^) and D6S291 (p = 0.045) (Table 3).

**Table 3:** Allelic distribution of STR markers.

Microsatellite	Allele	Frequency patients	Frequency controls	p	pc	Odds ratio [95%CI]
**D6S276**				**NS**		
**D6S265**				**0.02**	**0.26**	
	19	2.3	8.9	0.049	0.6	0.240 [0.052–1.109]
**MICA**				**NS**		
	5	5.7	17.9	0.01	0.06	0.280 [0.102–0.772]
**TNFb**				**0.009**	**0.063**	
	4	55.2	32.5	0.001	**0.007**	2.554 [1.449–4.500]
**TNFa**				**0.006**	**0.084**	
	11	21.8	10.6	0.025	0.35	2.364 [1.097–5.094]
**TNFc**				**0.000001**	**0.000002**	
	1	100	91.1	0.003	0.006	5.212 [0.793–34.269]
	2	21.8	53.7	0.00004	0.00008	0.241 [0.130–0.449]
**D6S273**				**NS**		
**D6S291**				**0.005**	**0.045**	
	12	12.6	27.6	0.009	0.08	0.379 [0.180–0.799]

NS: not significant.

p values indicated in front of the marker names correspond to the locus level analysis performed using the BIGDAWG package; pc were obtained after Bonferroni's correction.

According to the repetition number pattern, TNFb4 was significantly more frequent in patients than in HC, TNFc1 was also positively associated with SLE while TNFc2 was identified to be negatively associated with the disease (p<10^−5^, OR = 0.24). (Table 3)

After logistic regression all these markers remained associated with p value = 0.04 for TNFc1; 0.001 for TNFb4.

### 3.3. Clinical and serological associations with STR alleles

The majority of clinical and serological correlations were noted with TNF alleles (Table 4).

**Table 4:** Clinical and serological associations with STR markers.

STR marker	Parameters(n)	Positive	Negative	P**	Odds ratio [95%CI]
**TNFb4**	**LN(79)**	47.4%	68.3%	.060	0.418[0.167–01.044]
**TNFa11**	**LN(79)**	13.2%	31.7%	.050	0.326 [0.104–1.028]
**TNFc2**	**Anti-Sm(84)**	4.0%	30.5%	.009*	0.095 [0.012–0.756]
	**Anti-Cl(70)**	14.3%	39.3%	.017*	0.258 [0.082–0.813]
	**RF(57)**	50.0%	14.0%	.005*	6.167 [1.587–23.956]
**TNFc HET**	**Anti-Sm(84)**	5.3%	36.9%	.008*	0.095 [0.012–0.756]
	**RF(57)**	53.8%	15.9%	.005*	6.167 [1.587–23.956]
**MICA_5**	**Anti-RO52 (84)**	12.9%	0.0%	.016	1.148 [1.003–1.315]
**D6S265_12**	**C3 (67)**	6.5%	25.0%	.052	0.207 [0.041–1.045]
**D6S276_10**	**Anti-β2GP I (64)**	0.0%	30.4%	.026	0.696 [0.575–0.575]

HET:Heterozygous genotype, LN: lupus nephritis, RF: Rheumatoid factors, Cl: cardiolipine.

n: number of patients included in each analysis

* association remained significant after Bonferroni's correction.

**we included in this analysis significant associations or associations close to significance.

Patients with lupus nephritis expressed TNFb4 and TNFa11 more than patient without LN. The TNFc2 allele was negatively associated with the production of anti-Sm and anti-cardiolipin antibodies, however this allele, was more frequent in patients producing rheumatoid factors. (Table 4)

### 3.4. Haplotype analysis

The three- marker window covering the TNF region showed the strongest association with SLE in this study. Seven haplotypes were significantly increased in patients. After Bonferroni’s correction, four haplotypes lost their association with the disease (Table 5).

**Table 5:** Three-marker window haplotype analysis.

3-marker sliding window	Score global p	Significant haplotypes	Frequency case/control (%)	Pearson’s p	pc	Odds ratio [95%CI]
D6S276-D6S265-MICA	NS	17-11-6	3.7/0	0.03	NS	10.55 [1.1–102]
D6S265-MICA-TNFb	NS					
MICA-TNFb-TNFa	NS	5.1-3-2	11.5/3.6	0.003	0.02	2.20 [0.83–5.79]
TNFb-TNFa-TNFc	0.0003	4-11-1	10.26/0.8	0.00001	0.0001	19.34 [3.56–105]
		4-7-1	6.32/0.5	0.003	0.04	14.66 [1.8–118]
TNFa-TNFc-D6S273	0.007	11-1-16	7.2/1.63	0.003	0.03	4.3 [0.91–20.28]
		2-1-16	7.4/2.34	0.02	NS	2.67 [0.66–10.79]
TNFc-D6S273-D6S291	0.007	1-16-10	6.7/0	0.01	NS	1,6E+144
		1-16-14	6.3/1.4	0.04	NS	5.31 [0.86–32.6]

**NS:** not significant

When we included DR DQ HLA class II alleles in the analysis[7], we noted the existence of 2 associated regions. The most statistically significant encompassed the 3TNF markers; the latter covered the DR region (S2 Fig).

## 4. Discussion

Microsatellites in the HLA region are reported to be of particular interest in the susceptibility and pathogenesis of different immune mediated diseases[11–12]. As for systemic lupus erythematosus, no studies have been carried out in North African Lupus patients concerning HLA microsatellite markers.

We investigated in this study 8 STR covering the whole HLA region. D6S276 marker is telomeric to the class I region, and was found to be associated with numerous inflammatory diseases. D6S276 microsatellite is associated with pemphigus foliaceus in the Tunisian population [2]. In accordance with Shai et al [13], no significant difference could be established in D6S276 alleles distribution in our SLE patients when compared with healthy controls.

D6S265 marker also situated in HLA class I region does not seem to be implicated in SLE in our patients since the association found disappeared after Bonferroni’s correction (p = 0.02; pc = 0.26). This result was also reported by Smerdel-Ramoya et al [10].

The third marker, located within the MHC class I region, was MHC class I chain-related genes (MIC). In previous studies sequence analysis of the MIC-A gene showed a trinucleotide repeat (GCT) microsatellite polymorphism within the trans-membrane region. So far, seven alleles of the exon 5 of the MIC-A gene, which consist of 4, 5, 6, 8, 9 and 10 repetitions of GCT, or five repetitions of GCT with an additional nucleotide insertion (GGCT), have been identified. Recent works support the findings that MIC-A is associated with several autoimmune diseases [14].

In our study we tried to elucidate the role of MICA microsatellite polymorphism in SLE development. In accord with a Spanish case control study [15], we have found significant decrease of frequencies of the MICA-A5 allele in SLE patients. This allele was however reported to be predisposing to lupus in the Italian population [16]. The positive associations with MICA-A5.1 illustrated in these two studies did not appear in ours. MICA-A9 and MICA-A6 were reported to be protective in Italians and in Czechs respectively [16–17]. These negative associations have not been reported by others. In our study there were no statistic differences between patients and controls concerning MICA-A9 and A6.

The implication of MICA-A5 in the susceptibility of SLE can be explained by the findings of Yoshida et al. These researchers demonstrated that MICA 129Met-A5haplotype suppresses the expression of NKG2-D on NK cells, and thus inhibits NK cell cytotoxicity. This is consistent with previous findings showing that NK cell cytotoxicity is significantly decreased in SLE [18–19].

Gupta et al have reported the association between MICA gene polymorphism and autoantibody formation in type I diabetes [20]. The present study, to our knowledge, is the first one to show the association between MICA gene polymorphism and autoantibody formation (anti-Ro52) in SLE.

As we moved away from HLA class I region and covered that coding TNF, many associations between STR markers and SLE appeared. There were significant statistical differences in frequencies of TNF microsatellite markers TNFb4, a11, c1 and c2 between patients and healthy controls (p = 0.001, 0.025, 0.003 and 0.00004 respectively). TNFb4, a11 and c1 conferred susceptibility while TNFc2 was protective. Concerning these STR, conflicting reports exist in the literature. M Van der Linden reported similar findings: the presence of TNFa1 conferred susceptibility to develop SLE in Caucasian patients [21]. Naves et al. explored 4 STR markers in the TNF (a, b, c and d) in the coding region and demonstrated, contrarily to our findings, that TNF c2 was significantly increased in SLE Spanish patients [22].

D’Alfonso et al studied 3 polymorphisms in TNF coding region -238/A, -308/A and TNFa microsatellite. They concluded that TNF region do not seem to play a role in SLE susceptibility in the Italian population [23]. Lack of TNFA-308A association with SLE was later confirmed in Caucasian SLE families [24]. A larger Caucasian study suggested that TNFA-308A association with SLE was due to its linkage disequilibrium with low gene copy number of C4A and C4B [25].

Concerning associations between STR and clinical manifestations, the most relevant were noted with lupus nephritis. Patients carrying TNFb4 and a11 developed less kidney injury compared to those without. TNF c2 was protective and prevented development of anti-Sm and anti-cardiolipine antibodies and predisposed to rheumatoid factor production.

A.H. Hajeer et al reported a positive association between SLE and microsatellite markers TNFa2, b3 and d2 alleles. These three markers were significantly associated with photosensitivity and Raynaud's phenomenon as well as with anti-SSA antibodies production [26].

In the Greek population, TNF a11 frequencies were higher in SLE patients with renal disease and TNF a2 and b 3 frequencies in those without [27].

The last 2 STRs we investigated were D6S273 and D6S291. We found global significant difference only with D6S291. The allele 12 was negatively associated with SLE. This contrasts with the findings of Smerdel-Ramoya et al who demonstrated the association of D6S273with SLE in the Norwegian population. Unlike D6S273, D6S291 was not associated [10].

The use of sliding window approach of three contiguous markers in the haplotype analysis allowed us, to prove that two main SLE associated regions exist in our south Tunisian population. The most statistically significant is the region containing the 3 TNF STR markers and the second one containing the DRB1* locus. In fact, the lowest p value was found with the window containing TNF markers. Moreover, p value increased when moving away from the TNF region and decreased again when the DR marker was included.

The persistence of the significant association of D6S291 with SLE after logistic regression may be due to the existence of another associated marker beyond this STR, a hypothesis which should be investigated in further studies.

In conclusion, our results showed that specific alleles of five loci, in addition to the conventional DR/DQ, were found to be associated with SLE: D6S291, TNFa, TNFb, TNFc and D6S265. Interestingly, these findings indicate that the chromosomal region encompassing the TNF loci plays an important role in SLE probably by the induction of a high TNF production which increases inflammatory manifestations. However, more studies within other populations are necessary to evidence the general relevance of this polymorphism for SLE.

## Supporting information

S1 Fig Genetic map of the human MHC with microsatellites genotyped in this study. (TIF)

S2 Fig SLE patients versus healthy controls; global p-values for sub-haplotypes in the HLA region(1: p = 0.0002; 2: p = 0.007; 3: p = 0.002; 4: p = 0.02). (TIF)

S1 Table Characteristics of studied STR. (DOCX)

S2 Table Clinical and immunological manifestations in our patients. (DOCX)

S3 Table Allelic distribution of STR markers. (DOCX)

S4 Table Clinical and serological associations with STR markers. (DOCX)

S5 Table Three-marker window haplotype analysis. (DOCX)
