# Supplementary figures and images for: HLA Class III: A susceptibility region to systemic lupus erythematosus in Tunisian population
Source: PLoS One. 2018 Jun 18;13(6):e0198549. doi: 10.1371/journal.pone.0198549 (PMC6005577; doi:10.1371/journal.pone.0198549)

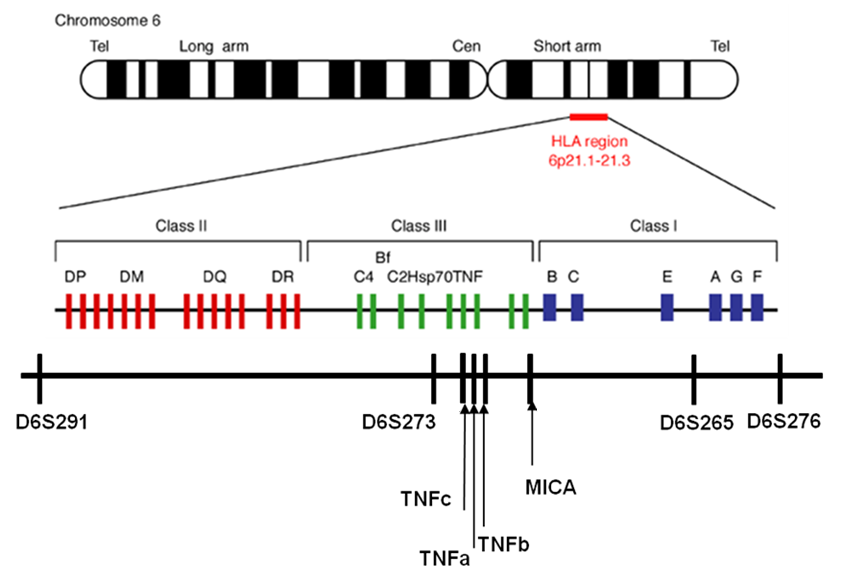

Supplement: S1 Fig — (TIF) [file pone.0198549.s001.tif]

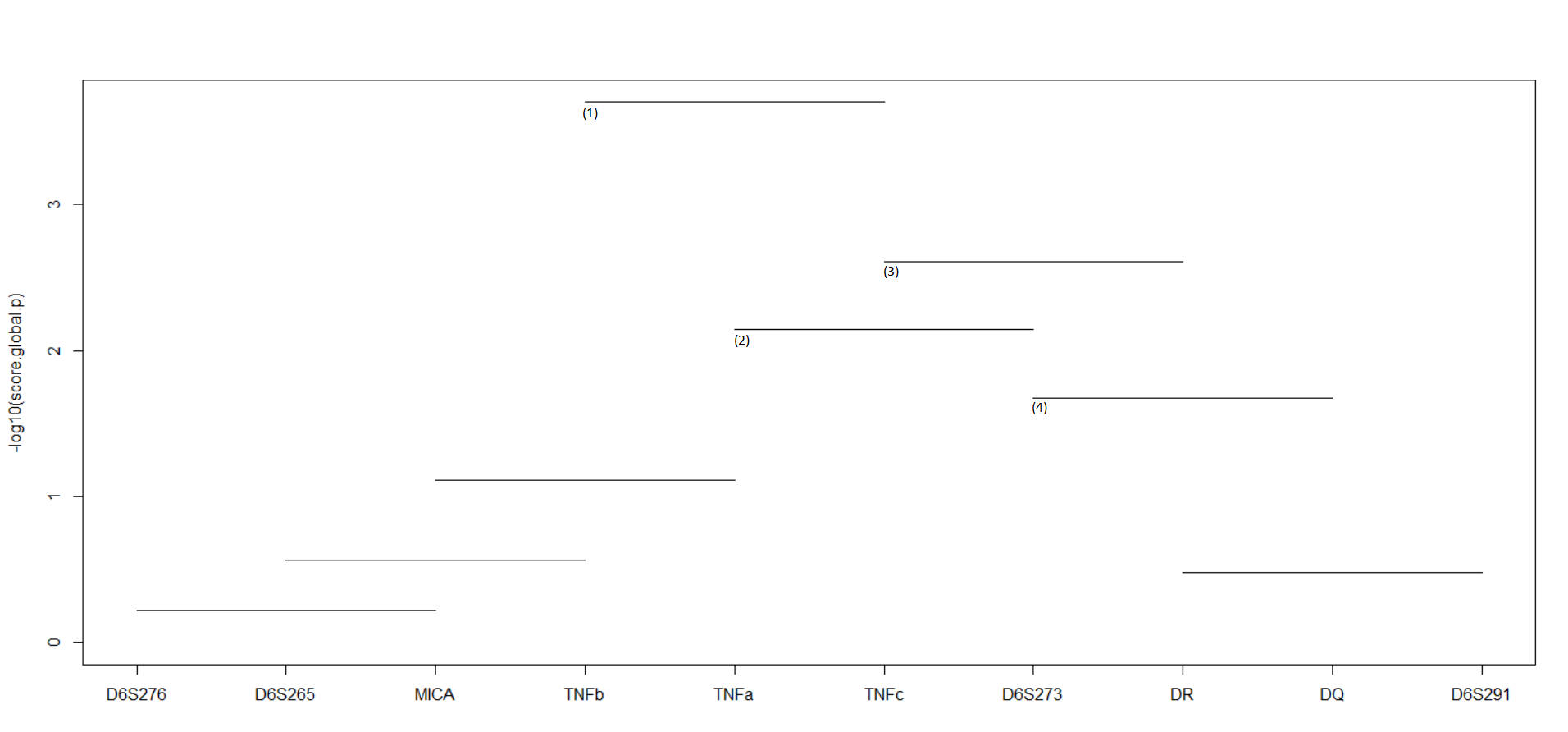

Supplement: S2 Fig — (TIF) [file pone.0198549.s002.tif]
