# Supplementary material for: HLA Class III: A susceptibility region to systemic lupus erythematosus in Tunisian population
Source: PLoS One. 2018 Jun 18;13(6):e0198549. doi: 10.1371/journal.pone.0198549 (PMC6005577; doi:10.1371/journal.pone.0198549)
Supplement: S1 Table — (DOCX) [file pone.0198549.s003.docx]

| ***STR*** | ***Localization*** | ***Repeats*** | ***Primers*** | ***Labeling*** |
| --- | --- | --- | --- | --- |
| ***D6S291*** | 6p21.2 ; 3,6-4cM cent DPB1 | CA | F :5’-CTCAGAGGATGCCATGTCTAA-3’  R :5’-GGGGATGACGAATTATTCACTAACT-3’ | 6-FAM- |
| ***D6S273*** | Hsp70-Bat2 (96 kb) Tel Hsp70 | CA | F:5’-GCAACTTTTCTGTCAATCCA-3’  R:5’-ACCAAACTTCAAATTTTCGG-3’ | 6-FAM- |
| ***TNFc*** | Intron of TNFB | TC | F :5’-GGTTTCTCTGACTGCATCTTGTCC-3’  R :5’-TCATGGGGAGAACCTGCAGAGAA-3’ | PET |
| ***TNFa*** | 6p21.3 Tel  (3,5 kb)/TNF B | AC | F:5’-GCCTCTAGATTTCATCCAGCCACA-3’  R:5’-CCTCTCCCCCTGCAACACACA-3’ | VIC |
| ***TNFb*** | 6p21.3 Tel. (3,5 kb)/TNF B | TC | F:5’-GCACTCCAGCCTAGGCCACAGA-3’  R:5’-GTGTGTGTTGCAGGGGAGAGAG-3’ | VIC |
| ***MICA*** | 40 Kb cent to HLA-B | GCT | F :5’-CCTTTTTTTCAGGGAAAGTGC-3’  R :5’-CCTTACCATCTCCAGAAACTGC-3’ | NED |
| ***D6S265*** | HLA-E/HLA-A | CA | F :5’-ACGTTCGTACCCATTAACCT-3’  R :5’-ATCGAGGTAAACAGCAGAAA-3’ | NED |
| ***D6S276*** | Tel (6500 kb) HLA-A | CA | F:5’-TCAATCAAATCATCCCCAGAAG-3’  R:5’-GGGTGCAACTTGTTCCTCCT-3’ | VIC |
