# Supplementary material for: HLA Class III: A susceptibility region to systemic lupus erythematosus in Tunisian population
Source: PLoS One. 2018 Jun 18;13(6):e0198549. doi: 10.1371/journal.pone.0198549 (PMC6005577; doi:10.1371/journal.pone.0198549)
Supplement: S2 Table — (DOCX) [file pone.0198549.s004.docx]

|  | **Number** | **Frequency%** | **Availability** |
| --- | --- | --- | --- |
| **Sex F/M** | 76/11 |  |  |
| **Clinical manifestations** |  |  |  |
| Malar rush | 41 | 51.9 | 90.8 |
| Photosensitivity | 36 | 45.6 | 90.8 |
| Buccal ulceration | 11 | 13.9 | 90.8 |
| Anemia | 66 | 48.6 | 89.7 |
| Arthritis | 18 | 22.8 | 90.8 |
| Polyathralgia | 51 | 64.6 | 90.8 |
| Lupus nephritis | 38 | 48.1 | 90.8 |
| Pericarditis | 19 | 24.1 | 90.8 |
| Pleurisy | 12 | 15.2 | 90.8 |
| Raynaud's syndrome | 6 | 7.6 | 90.8 |
| Thrombosis | 13 | 17.1 | 87.4 |
| Neurologic disorders | 12 | 16.2 | 85.1 |
| **Serology** |  |  |  |
| Anti-dsDNA | 60 | 70.6 | 97.7 |
| Anti-nucleosome | 51 | 60.7 | 96.6 |
| Anti-Sm | 25 | 29.8 | 96.6 |
| Anti-RNP | 25 | 29.8 | 96.6 |
| Anti-SSA | 48 | 57.1 | 96.6 |
| Anti-SSB | 20 | 23.8 | 96.6 |
| Anti-Ribosome | 16 | 19 | 96.6 |
| Anti-Histone | 27 | 32.1 | 96.6 |
| Anti RO52 | 31 | 36.9 | 96.6 |
| Low CH50 | 36 | 60 | 69 |
| Low C3 | 31 | 46.3 | 77 |
| Low C4 | 34 | 50.7 | 77 |
| Anti-cardiolipin | 42 | 60 | 80.5 |
| Anti-β2gpI | 23 | 35.9 | 73.6 |
| Rheumatoid factors | 14 | 24.6 | 65.5 |

F: female, M: male
