# Supplementary material for: HLA Class III: A susceptibility region to systemic lupus erythematosus in Tunisian population
Source: PLoS One. 2018 Jun 18;13(6):e0198549. doi: 10.1371/journal.pone.0198549 (PMC6005577; doi:10.1371/journal.pone.0198549)
Supplement: S3 Table — (DOCX) [file pone.0198549.s005.docx]

| **Microsatellite** | **Allele** | **Frequencypatients** | **Frequency controls** | **p** | **pc** | **Odds ratio [95%CI]** |
| --- | --- | --- | --- | --- | --- | --- |
| ***D6S276*** |  |  |  | **NS** |  |  |
| ***D6S265*** |  |  |  | **0.02** | **0.26** |  |
|  | 19 | 2.3 | 8.9 | 0.049 | 0.6 | 0.240 [0.052-1.109] |
| ***MICA*** |  |  |  | **NS** |  |  |
|  | 5 | 5.7 | 17.9 | 0.01 | 0.06 | 0.280 [0.102-0.772] |
| ***TNFb*** |  |  |  | **0.009** | **0.063** |  |
|  | 4 | 55.2 | 32.5 | 0.001 | **0.007** | 2.554 [1.449-4.500] |
| ***TNFa*** |  |  |  | **0.006** | **0.084** |  |
|  | 11 | 21.8 | 10.6 | 0.025 | 0.35 | 2.364 [1.097-5.094] |
| ***TNFc*** |  |  |  | **0.000001** | **0.000002** |  |
|  | 1 | 100 | 91.1 | 0.003 | 0.006 | 5.212 [0.793-34.269] |
|  | 2 | 21.8 | 53.7 | 0.00004 | 0.00008 | 0.241 [0.130-0.449] |
| ***D6S273*** |  |  |  | **NS** |  |  |
| ***D6S291*** |  |  |  | **0.005** | **0.045** |  |
|  | 12 | 12.6 | 27.6 | 0.009 | 0.08 | 0.379 [0.180-0.799] |

NS: not significant

p values indicated in front of the marker names correspond to the locus level analysis performed using the BIGDAWG package;pc were obtained after Bonferroni's correction
