# Supplementary material for: HLA Class III: A susceptibility region to systemic lupus erythematosus in Tunisian population
Source: PLoS One. 2018 Jun 18;13(6):e0198549. doi: 10.1371/journal.pone.0198549 (PMC6005577; doi:10.1371/journal.pone.0198549)
Supplement: S4 Table — (DOCX) [file pone.0198549.s006.docx]

| **STR marker** | **Parameters (n)** | **Positive** | **Negative** | **P**** | **Oddsratio [95%CI]** |
| --- | --- | --- | --- | --- | --- |
| **TNFb4** | **LN (79)** | 47.4% | 68.3% | .060 | 0.418[0.167-01.044] |
| **TNFa11** | **LN (79)** | 13.2% | 31.7% | .050 | 0.326 [0.104-1.028] |
| **TNFc2** | **Anti-Sm (84)** | 4.0% | 30.5% | .009* | 0.095 [0.012-0.756] |
|  | **Anti-Cl (70)** | 14.3% | 39.3% | .017* | 0.258 [0.082-0.813] |
|  | **RF (57)** | 50.0% | 14.0% | .005* | 6.167 [1.587-23.956] |
| **TNFc HET** | **Anti-Sm (84)** | 5.3% | 36.9% | .008* | 0.095 [0.012-0.756] |
|  | **RF (57)** | 53.8% | 15.9% | .005* | 6.167 [1.587-23.956] |
| **MICA_5** | **Anti-RO52 (84)** | 12.9% | 0.0% | .016 | 1.148 [1.003-1.315] |
| **D6S265_12** | **C3 (67)** | 6.5% | 25.0% | .052 | 0.207 [0.041-1.045] |
| **D6S276_10** | **Anti-β2GP I (64)** | 0.0% | 30.4% | .026 | 0.696 [0.575-0.575] |

HET:Heterozygous genotype, LN: lupus nephritis, RF: Rheumatoid factors, Cl: cardiolipine
n : number of patients included in each analysis; * association remained significant after Bonferroni's correction; **we included in this analysis significant associations or associations close to significance
