# Supplementary material for: HLA Class III: A susceptibility region to systemic lupus erythematosus in Tunisian population
Source: PLoS One. 2018 Jun 18;13(6):e0198549. doi: 10.1371/journal.pone.0198549 (PMC6005577; doi:10.1371/journal.pone.0198549)
Supplement: S5 Table — (DOCX) [file pone.0198549.s007.docx]

| **3-marker sliding window** | **Score global p** | **Significant haplotypes** | **Frequency case/control (%)** | **Pearson’s p** | **pc** | **Odds ratio [95%CI]** |
| --- | --- | --- | --- | --- | --- | --- |
| D6S276-D6S265-MICA | NS | 17-11-6 | 3.7/0 | 0.03 | NS | 10.55 [1.1-102] |
| D6S265-MICA-TNFb | NS |  |  |  |  |  |
| MICA-TNFb-TNFa | NS | 5.1-3-2 | 11.5/3.6 | 0.003 | 0.02 | 2.20 [0.83-5.79] |
| TNFb-TNFa-TNFc | 0.0003 | 4-11-1 | 10.26/0.8 | 0.00001 | 0.0001 | 19.34 [3.56-105] |
|  |  | 4-7-1 | 6.32/0.5 | 0.003 | 0.04 | 14.66 [1.8-118] |
| TNFa-TNFc-D6S273 | 0.007 | 11-1-16 | 7.2/1.63 | 0.003 | 0.03 | 4.3 [0.91-20.28] |
|  |  | 2-1-16 | 7.4/2.34 | 0.02 | NS | 2.67 [0.66-10.79] |
| TNFc-D6S273-D6S291 | 0.007 | 1-16-10 | 6.7/0 | 0.01 | NS | 1.6E+144 |
|  |  | 1-16-14 | 6.3/1.4 | 0.04 | NS | 5.31 [0.86-32.6] |

**NS:** not significant
